# Lysine Acetyltransferase 8: A Target for Natural Compounds in Cancer Therapy

**DOI:** 10.3390/ijms26115257

**Published:** 2025-05-29

**Authors:** Lei Wang, Liting Zhao, Xintian Lan, Ming Zhu, Yiying Tan, Haoming Luo, Donglu Wu

**Affiliations:** 1 School of Pharmacy, Changchun University of Chinese Medicine, Changchun 130117, China; 13578702468@163.com (L.W.); zlt15020747022@163.com (L.Z.); lanxintian2022@163.com (X.L.); zuming124@163.com (M.Z.); 2 Jilin Ginseng Academy, Changchun University of Chinese Medicine, Changchun 130117, China; t2589954433@163.com; 3 School of Clinical Medical, Changchun University of Chinese Medicine, Changchun 130117, China

**Keywords:** KAT8, natural compounds, cancer, H4K16ac, targeted therapeutics

## Abstract

Lysine acetyltransferase 8 (KAT8) is a member of the MYST family of histone acetyltransferases. It catalyzes the acetylation of histone H4 at lysine 16 (H4K16ac) and non-histone proteins. Abnormal upregulation or downregulation of KAT8 and its associated H4K16ac have been observed in malignant tumors, suggesting its close association with tumorigenesis and progression. Characterized by structural diversity and multi-target mechanisms, natural agents have been increasingly shown to possess significant antitumor activity. This review focuses on KAT8, summarizing its molecular mechanisms in regulating tumor development by catalyzing substrate protein acetylation, which impacts tumor cell proliferation, cell cycle regulation, apoptosis, DNA damage repair, and autophagy. It also systematically discusses the pharmacological activities and molecular mechanisms of small-molecule agents that target KAT8 to inhibit tumor proliferation, including natural compounds, synthetic drugs, and non-coding RNAs.

## 1. Introduction

Malignant tumors are characterized by high incidence and mortality and poor prognosis worldwide [1]. Current clinical strategies combine early detection with treatments such as surgery, chemotherapy, and immunotherapy to improve patient survival. However, therapeutic efficacy depends on multiple factors, including tumor type and drug resistance [2]. Natural medicines—encompassing animal-, plant-, mineral-, and marine-derived agents validated by modern pharmaceutical systems—exhibit pharmacological activity. Bioactive natural compounds extracted from these sources are characterized by their structural diversity and multi-target mechanisms. In recent years, numerous natural compounds have demonstrated potent antitumor activity and are utilized as chemotherapeutic agents (e.g., paclitaxel) or adjuvant therapies in cancer treatment [3].

**Figure 1:**
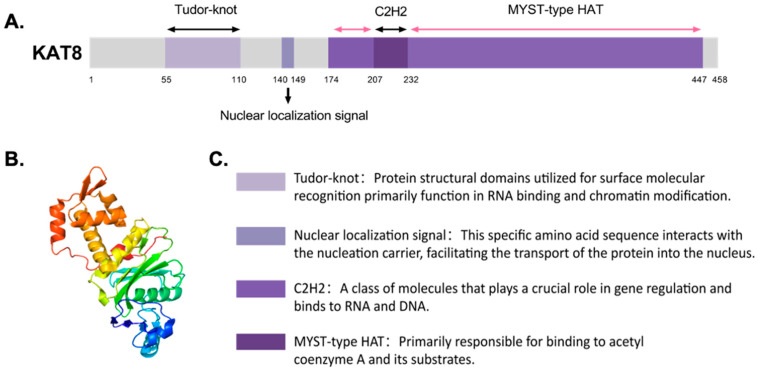
Three-dimensional structure and functional structural domains of KAT8. (**A**) Functional structural domains. (**B**) 3D structure of crystal structure of MYST acetyltransferase domain in complex with N-(1-(5-bromo-2-methoxyphenyl)-1H-1,2,3-triazol-4-yl)-2-methoxybenzenesulfonamide [7]. (**C**) Legend of each structural domain and its corresponding function.

Lysine acetyltransferase 8 (KAT8), formerly called males absent on the first (MOF) and MYST histone acetyltransferase 1 (MYST1), belongs to the MYST family of HATs. Initially identified in Drosophila as a component of the X-chromosome dosage compensation complex, KAT8 balances X-encoded protein/enzyme levels between sexes [4]. It is evolutionarily conserved across species, including humans. Structurally, KAT8 contains a chromodomain, a zinc finger domain, and a canonical MYST (MOZ/SAS) domain (Figure 1) [5]. The MYST (HAT) domain contains an acetyl-coenzyme A binding site and a histone-binding region, while the C2HC-type zinc finger domain is critical for substrate recognition [6].

KAT8 exhibits broad substrate specificity, catalyzing acetylation of histone 4 at lysine 16 (H4K16) and non-histone proteins [8]. KAT8 regulates diverse cellular processes through its catalytic activities, including embryonic stem cell pluripotency, gene transcription, DNA damage repair, cell proliferation, apoptosis, autophagy, and mitochondrial homeostasis [9]. In non-small cell lung cancer (NSCLC) cells, KAT8 upregulates p27 transcription by enhancing H4K16 acetylation (H4K16ac) in the S-phase kinase-associated protein 2 (SKP2) promoter, promoting S-phase progression and proliferation [10]. KAT8 serves as the catalytic subunit in two distinct complexes: the male-specific lethal (MSL) and non-specific lethal (NSL) complexes. Beyond the catalytic subunit KAT8, the MSL complex contains MSL1, MSL2, and MSL3 subunits that specifically acetylate lysine 16 on histone H4 [11]. In contrast, the NSL complex (comprising KAT8 regulatory NSL complex subunits 1 (*KANSL1*), 2 (*KANSL2*), 3 (*KANSL3*), PHD finger protein 20 (PHF20), host cell factor C1 (*HCFC1*), WD repeat domain 5 (*WDR5*), O-linked N-acetylglucosamine transferase (OGT), microspherule protein 1 (MCRS1)) exhibits broader substrate specificity, acetylating histone H4 at lysines 5, 8, and 18, in addition to H4K16, to regulate diverse biological functions [12]. Moreover, KAT8 acetylates non-histone proteins such as tumor protein p53 (TP53), lysine demethylase 1A (KDM1A/LSD1), and interferon regulatory factor 3 (*IRF3*) [13]. For example, KAT8 acetylates p53 at lysine 120 (K120) in its DNA-binding domain, modulating DNA damage responses [14]. YEATS domain-containing protein 4 (YEATS4), a conserved nuclear protein overexpressed in multiple cancers, was acetylated by KAT8 in bladder cancer cells, disrupting its interaction with HECT, UBA, and WWE domain containing E3 ubiquitin protein ligase 1 (HUWE1), inhibiting its ubiquitination and degradation, thereby suppressing tumor proliferation [15,16]. Selenoprotein P 1 (SEPP1), a member of the selenoprotein family of selenium transporter proteins and antioxidant enzymes, with a cluster of differentiation 8 (CD8)^+^ T cell abundance, could be acetylated by KAT8 at lysines 247 and 249, which enhanced CD8^+^ T cell activity and antitumor immunity in pancreatic cancer [17,18]. Dysregulated KAT8 expression or enzymatic activity is implicated in neurological disorders [19], immune diseases [20], and cancer [21]. This review summarizes the roles of KAT8 in tumorigenesis and progression and the natural compounds and small-molecule drugs targeting KAT8 for cancer therapy.

## 2. Aberrant Expression of Lysine Acetyltransferase 8 (KAT8) in Cancer

Numerous studies have revealed that KAT8 and H4K16ac are intricately associated with tumor initiation, development, and progression. Acetylation that occurs at H4k16 reduces the negative charge of histones, weakening their interaction with DNA and thereby relaxing chromatin, which facilitates DNA damage repair, gene transcription, and recombination [22]. It is reported that upregulation of H4K16ac promotes the proliferation of NSCLC and malignant glioma cells [23]. In addition, H4K16 and α-tubulin deacetylation mediated by HDAC6 promotes correct spindle organization and meiotic apparatus assembly during porcine oocyte maturation, indicating the role of H4K16ac in regulating the cell cycle of cancer cells [24]. Moreover, the repression of H3K18 and H4K16 acetylation at the proto-oncogene Myc promoter region inhibits the Pol II recruitment to initiate Myc transcription in breast cancer cells [25]. Therefore, the imbalance of H4K16ac and its catalytic enzyme KAT8 is tightly related with tumorigenesis.

Downregulation of KAT8 and its associated H4K16ac has been observed in renal cell carcinoma, ovarian cancer, hepatocellular carcinoma (HCC), and gastric cancer [26]. Specifically, KAT8 expression was reduced by over 75% in 41% of patients with primary breast cancer and 79% of patients with medulloblastoma. In a study analyzing 5102 medulloblastoma tissues, both mRNA and protein levels of KAT8 were markedly reduced compared to normal tissues, accompanied by decreased H4K16ac levels. Notably, patients with low KAT8 expression exhibited significantly lower survival than those with high expression [27]. Similarly, KAT8 expression was downregulated in 90.5% (19/21) of patients with renal cell carcinoma [28], and its levels correlated positively with survival in patients with liver and gastric cancer [29].

**Figure 2:**
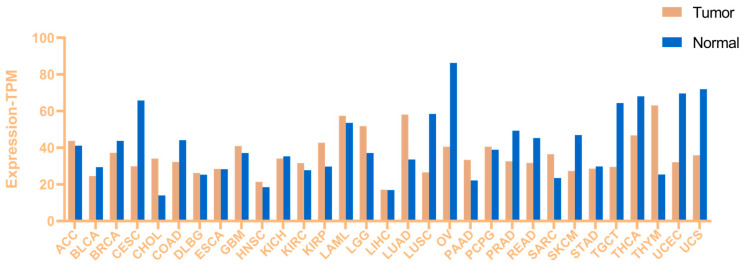
Differential expression of KAT8 in indicated tumor and normal tissues (TCGA database updated by 21 May 2025).

Conversely, KAT8 is aberrantly upregulated in certain malignancies, including glioblastoma, oral tongue squamous cell carcinoma, NSCLC, thymic lymphoma, endometrial cancer, and thyroid cancer [30,31]. For example, KAT8 mRNA levels were 1.5–2 times higher in glioma tissues than in normal brain tissues (*p* < 0.05) [32]. The differential expression patterns of KAT8 across tumor and normal tissues, summarized in Figure 2 based on The Cancer Genome Atlas (TCGA) database, suggest context-dependent roles of KAT8 in high- versus low-expression tumors.

## 3. The Role of Lysine Acetyltransferase 8 (KAT8) in Cancer Progression

As a core member of the MYST HAT family, KAT8 dynamically regulates chromatin remodeling and gene transcription through H4K16 acetylation, while its acetylation of non-histone proteins modulates their activity, thereby influencing critical cellular processes. It is evident that dysregulation of KAT8 expression or enzymatic activity is closely linked to tumorigenesis. Paradoxically, however, KAT8 is overexpressed in some cancers but underexpressed in others, highlighting its divergent roles in tumor proliferation, apoptosis, and autophagy.

### 3.1. Lysine Acetyltransferase 8 (KAT8) and Apoptosis

Apoptosis, also called programmed cell death, is a genetically regulated process of autonomous and orderly cell death that maintains intracellular homeostasis [33]. In tumors with low KAT8 expression, KAT8 promotes apoptosis. For example, nuclear protein 1 transcriptional regulator (*NUPR1*) overexpression-mediated downregulation of KAT8 inhibited programmed apoptosis in HCC cells [34]. Consistent with this, KAT8 overexpression increased H4K16ac levels, reduced carbonic anhydrase 9 (CA9) expression, and promoted apoptosis in clear cell renal cell carcinoma [35]. Moreover, KAT8 regulated p53 K120 acetylation to induce apoptosis in H9C2 cardiomyocytes, U2OS osteosarcoma cells, and MCF-7 breast cancer cells [36,37]. Mechanistically, KAT8-mediated p53 K120 acetylation promotes p53 recruitment to the promoters of pro-apoptotic genes BCL2-associated X apoptosis regulator (*BAX*) and BCL2 binding component 3 (*BBC3/PUMA*), enhancing their transcription and inducing apoptosis in H9C2 cells [14]. In MCF-7 and MDA-MB231 breast cancer cells, small interfering RNA-mediated KAT8 knockdown reduced global H4K16ac levels, silenced the pro-apoptotic gene *PYD* and *CARD* domain containing (*PYCARD/TMS1/ASC*), and inhibited apoptosis [38].

It is reported that KAT8 autoacetylated at K274 residue, which could be deacetylated by sirtuin 1 (SIRT1). Overexpressed SIRT1 leads to downregulated KAT8 acetylation as well as enzyme activity, whereas it increases the recruitment to chromatin in Hela cells [39]. In HeLa cells, SIRT1 orchestrates an epigenetic-proteostatic cascade through physical interaction with histone acetyltransferases KAT8 and Tip60 (KAT5). This protein complex facilitates deacetylation of KAT8/Tip60, thereby abrogating their intrinsic HAT activity. The resultant suppression of DNA damage repair machinery culminates in apoptosis induction [40]. Moreover, it is reported that acetylation of SIRT1 promoted KAT8 ubiquitination dependent degradation [41]. KAT8 also bound to the SIRT1 promoter to enhance SIRT1 expression, which downregulated the signal transducer and activator of transcription 3 (*STAT3*) and promoted apoptosis in HepG2 cells [42]. In prostate cancer cells, KAT8 is able to catalyze the level of H4K16 acetylation modification in the promoter region of nuclear factor-κ B (*NF-κB*) to enhance its transcriptional activity, whereas the activation of NF-κB promotes the deacetylation of KAT8 by SIRT1 to downregulate the level of H4K16ac [43]. In addition, KAT8 repression induces Caspase 3 cleavage and AR-lacking PC3 cells apoptosis, which indicated that the balance of KAT8 acetylation mediated by KAT8 and SIRT1 is dependent on NF-κB activation and corelated with cancer cell apoptosis.

Conversely, in tumors with high KAT8 expression, KAT8 exhibits anti-apoptotic effects. For example, interferon-gamma (*IFNG*) stimulation induced the formation of KAT8-IRF1 transcriptional condensates in NSCLC cells, upregulating the CD274 molecule (*CD274/PD-L1*) to inhibit A549 cell apoptosis [42]. Pyruvate kinase M1/2 (*PKM/PKM2*), a glycolytic regulator, interacted with and phosphorylated Bcl-2 apoptosis regulator (*Bcl-2*) to upregulate its expression [44], suppressing apoptosis in glioblastoma cells and promoting NSCLC cell proliferation [45]. KAT8 also interacted with PKM2 in the nucleus to acetylate it at lysine 433, enhancing glycolysis and A549 cell proliferation [46]. Consistent with this, KAT8 deficiency promoted apoptosis in BHP-10-3 and TT2609 thyroid cancer cells [47].

In summary, KAT8 bidirectionally regulates apoptosis through interactions with apoptotic factors and signaling pathways. Its role in cancer cell apoptosis depends on its role in cancer progression, as reflected by the KAT8 expression level.

### 3.2. Lysine Acetyltransferase 8 (KAT8) and Cell Proliferation

One of the core biological characteristics of life, cell proliferation refers to the process by which cells generate new cells through division [48]. Multiple studies have revealed that KAT8 exerts distinct regulatory effects on tumor cell proliferation depending on its expression level (high/low) in tumor tissues [10].

In tissues and cells with low KAT8 expression, KAT8 typically suppresses proliferation. For example, KAT8 knockout induced hyperproliferation in MCF-7 breast cancer cells [49]. Research indicates that KAT8 inhibits the tumorigenicity of HCC cells, suppressing their proliferation both in vitro and in vivo [33]. Consistent with this, KAT8 expression was downregulated in HCC tissue samples and correlated positively with estrogen receptor 1 (*ESR1/ERα*). Targeted knockdown of KAT8 upregulated endogenous ERα, downregulating nuclear receptor subfamily 0 group B member 2 (*NR0B2/SHP*) and SMAD family member 7 (*SMAD7)* expression and significantly promoting HCC cell proliferation [13]. Conversely, elevated KAT8 expression in MGC-803 gastric cancer cells increased H4K16ac levels, leading to cell cycle arrest at the G1 phase [50].

Whereas, in tissues and cells with high KAT8 expression, KAT8 often promotes proliferation. For example, KAT8 increased H4K16ac levels in the promoter region of the human papillomavirus oncoprotein E7 in cervical cancer cells, enhancing transcriptional activity and accelerating proliferation [51]. In endometrial cancer cells, the estrogen/estrogen receptor upregulates KAT8 expression, activates the phosphoinositide 3-kinase (PI3K)/protein kinase B (AKT) and Ras/Raf/mitogen-activated protein kinase (MEK)/extracellular signal-regulated kinase (ERK) pathways to inhibit apoptosis of Ishikawa uterine cancer cells [52]. Histone methyltransferase enhancer of zeste 2 polycomb repressive complex 2 subunit (*EZH2*) catalyzed the trimethylation of histone H3 at lysine 27 (H3K27me3), enhancing the transcriptional activity of polycomb repressive complex 2 and thereby suppressing the progression of malignancies such as breast, bladder, and endometrial cancers [53]. In tongue squamous cell carcinoma tissues, KAT8 expression was upregulated and correlated positively with EZH2 levels. The KAT8 inhibitor CHI-KAT8i5 downregulated KAT8, reduced EZH2 expression, and inhibited the tumorigenicity of UM1 cells [54]. NF-E2-related factor 2 (*NRF2*), a master regulator of cellular antioxidant responses, is able to regulate cellular resistance to oxidative stress, DNA damage repair, etc. and further regulates cancer progression [55]. KAT8 acetylates NRF2 at lysine 588, facilitating its nuclear translocation and the activation of downstream oncogenes such as NAD(P)H:quinone oxidoreductase 1 (*NQO1*) and heme oxygenase 1 (HMOX1/HO-1), promoting NSCLC proliferation. Specifically, KAT8 knockdown in vivo and in vitro suppressed NSCLC cell proliferation by reducing NRF2 acetylation [56]. KAT8-mediated acetylation of sirtuin 6 (SIRT6) significantly inhibited its deacetylase activity and disrupted its interaction with forkhead box A2 (*FOXA2*), leading to the transcriptional upregulation of zinc finger E-box binding homeobox 2 (*ZEB2*) [57]. Elevated ZEB2 expression correlated with poor overall survival in patients with cancer and tumor progression, promoting proliferation in A549 and H1299 NSCLC cells [58]. In thyroid cancer tissues with high KAT8 expression, KAT8 knockdown decreased the levels of cyclin D1 (CCND1) and D3 (CCND3), critical regulators of the G1/S transition, resulting in G1-phase cell cycle arrest and suppressed thyroid cancer cell proliferation [59]. Conversely, silencing KAT8 in malignant glioma cells downregulated cyclin-dependent kinase 1 (CDK1), cyclin A, and cyclin B expression and upregulated p21 expression, leading to G2/M-phase arrest [19]. While KAT8 is overexpressed in both thyroid cancer and malignant glioma tissues, the mechanisms underlying its cell cycle regulation differ between these two cancer types, which we speculate may be attributed to the diversity of KAT8 substrates involved in fulfilling the biological functions of different cell types.

In summary, KAT8 regulates tumor cell proliferation by modulating the cell cycle, tumorigenicity, and related pathways. Similar to its role in apoptosis, its regulatory effects vary across cancer types and are closely associated with its aberrant upregulation or downregulation in corresponding tumor tissues. This functional duality may stem from imbalances in KAT8 expression and catalytic activity.

### 3.3. Lysine Acetyltransferase 8 (KAT8) and DeoxyriboNucleic Acid Damage and Repair

Abnormal DNA damage repair in cells can lead to DNA mutations, compromising genomic stability and mediating the transition of cells from a homeostatic state to a malignant phenotype [60]. Compared to normal tissues, tumor tissues exhibit increased DNA damage, hindering DNA replication and elevating the incidence of DNA damage, particularly DNA double-strand breaks (DSBs) [61]. Dysregulated DNA replication control and DNA damage induce replication stress, a source of genomic instability and a hallmark of precancerous and cancerous cells [62]. The efficiency of DNA damage repair determines the subsequent progression of tumors.

It has been reported that KAT8 and its associated H4K16 acetylation play critical roles in DNA damage response and repair processes, including homologous recombination and DSB repair [63]. Knockout/overexpression of KAT8 correlated positively with phosphorylation of serine 139 in H2A.X variant histone (H2AX), a DNA damage marker (γH2AX), in MLL-AF9 leukemia cells, indicating that KAT8 promotes DNA damage repair and inhibits apoptosis in AF9 cells [64]. Consistent with this, the jumonji domain containing 6 arginine demethylase and lysine hydroxylase (JMJD6)-mediated upregulation of KAT8 in U2OS and MCF-7 cells enhanced H4K16ac levels near DSBs, facilitating DNA damage repair [65]. In RKO colon cancer cells, after DNA damage induction by ionizing radiation, ATM serine/threonine kinase (ATM) phosphorylated threonine 392 of KAT8, which recruited the mediator of DNA damage checkpoint protein 1 (MDC1) and BRCA1 DNA repair associated (*BRCA1*) to DNA damage sites for repair, thereby promoting colon cancer cell proliferation [66,67]. Proliferating cell nuclear antigen (PCNA), a DNA polymerase-interacting protein, participates in DNA replication and damage repair [68]. Studies have shown that KAT8 interacts with PCNA, regulating PCNA ubiquitination to promote its recruitment to replication stress-induced DNA damage sites, thereby initiating the translesion DNA synthesis pathway for DNA repair [69]. Notably, in U2OS osteosarcoma cells, inhibiting the interaction between PCNA and DNA polymerase η (POLH) blocks DNA damage repair and suppresses U2OS cell proliferation [70]. These findings suggest that KAT8 promotes osteosarcoma cell proliferation by regulating DNA damage repair via PCNA. The pivotal role of KAT8 in DNA damage repair highlights its potential as a therapeutic target. Inhibiting the acetyltransferase activity of KAT8 could suppress the repair of elevated DNA damage in tumor cells, offering a strategy for developing chemotherapeutic agents or adjuvant therapies for radiotherapy/chemotherapy.

### 3.4. Lysine Acetyltransferase 8 (KAT8) and Autophagy

Autophagy is a lysosome-dependent process that degrades aged, damaged, or excess organelles and proteins to maintain cellular homeostasis [71]. The role of autophagy in cancer varies depending on its stage, mutation type, and microenvironment [72]. During early tumorigenesis, autophagy acts as a survival pathway to suppress tumor initiation and progression [73]. However, in established tumors, autophagy protects cancer cells [74], while excessive activation induces autophagic cell death and inhibits tumor progression [75]. Numerous studies have revealed that post-translational modifications, particularly acetylation, play crucial roles in autophagy regulation [76].

In fibroblasts, KAT8 increased H4K16ac levels to promote the expression of the autophagy regulator transforming growth factor (*TGF*)-β, thereby inducing autophagy and inhibiting fibroblast differentiation [77]. Microtubule-associated protein 1 light chain 3 alpha (*MAP1LC3A/LC3*) is a key autophagy protein. During autophagy, its cytosolic form *(LC3-I*) is converted to the phosphatidylethanolamine-conjugated form (*LC3-II*), which localizes to autophagosomal membranes [78]. Rapamycin suppressed KAT8 levels and H4K16ac levels, promoting the conversion from LC3-I to LC3-II and activating autophagy in HeLa cervical cancer and U1810 NSCLC cells [79]. GABA type A receptor-associated protein-like 1 (GABARAPL1) is critical for the autophagolysosomal degradation pathway [80]. It inhibited the perinuclear transport of autophagosomes and lysosomes, induced lysosomal degradation, reduced autophagic flux, and regulated autophagy-related processes in breast cancer and LNCaP prostate cancer cells [81,82,83,84]. KAT8 acetylates H4K16 in the GABARAPL1 promoter region, inducing its expression and activating epidermal cell (HaEpi) autophagy [85]. Therefore, KAT8 may regulate autophagy in breast and prostate cancers via GABARAPL1. Additionally, treating neuroblastoma cells with MG149, a KAT8 inhibitor, impairs global H4K16 acetylation and autophagy regulator PTEN-Induced Kinase 1 (PINK1) expression of neuroblastoma cells further inhibiting autophagy receptor p62 recruitment, resulting in autophagy [86]. Moreover, PINK1 silence downregulates the autophagy receptor p62, inhibiting mitophagy and promoting renal cancer cell proliferation [87], which suggests that the role of KAT8 in activating the PINK1 signaling pathway could induce cancer cell autophagy and influence tumor cell proliferation.

### 3.5. Lysine Acetyltransferase 8 (KAT8) and Invasion/Migration

Tumor cells actively degrade the basement membrane and extracellular matrix to invade surrounding tissues, leading to cancer metastasis [88,89]. Knocking out KAT8 in HCC cells or silencing KAT8 in GBM cells significantly reduced their invasion and migration capabilities [88,89]. Similarly, upregulating KAT8 promoted the proliferation, migration, and invasion of endometrial cancer Ishikawa cells. In oesophageal squamous cell carcinoma (ESCC) cells, KAT8 catalyzed the acetylation of fascin actin-bundling protein 1 (FSCN1) at lysine 41, enhancing its F-actin-bundling activity and promoting filopodia/invadopodia formation to drive ESCC cell invasion [90]. Ubiquitin-specific peptidase 10 (USP10) stabilized KAT8 and increased H4K16 acetylation in the promoter region of annexin A2 (*ANXA2*), activating Wnt/β-catenin (*CTNNB1*) signaling to promote ESCC cell migration and invasion [91]. The tyrosine kinase bromodomain adjacent to zinc finger domain 1B (*BAZ1B/WSTF*) is upregulated in multiple cancers to drive migration and proliferation [92]. In MDA-MB-231 breast cancer cells, KAT8 acetylated WSTF at lysine 426, promoting its phosphorylation at serine 158 and thereby enhancing its kinase and transcriptional activity, driving breast cancer cell proliferation, migration, and invasion [93].

Epithelial-mesenchymal transition (EMT) is a process in which cells lose epithelial features and gain mesenchymal features. It is involved in embryogenesis, wound healing, and cancer progression, including metastasis [94,95]. Keratin 8 (KRT8), a major intermediate filament component, is closely associated with cell migration, invasion, and EMT [96]. In A549 NSCLC cells, KAT8 interacted with and acetylated LSD1, inhibiting LSD1 activity, which increased the methylation of histone H3 at lysine 4 in the promoters of KRT8 and cadherin 1 (*CDH1/E-cadherin*), upregulating their expression and promoting EMT and tumor invasion [97].

These findings demonstrate that KAT8 and its H4K16ac-mediated regulatory mechanisms are critically involved in tumorigenesis (Table 1).

## 4. Small-Molecule Drugs Targeting Lysine Acetyltransferase 8 (KAT8)

Small-molecule drugs are organic or synthetic compounds with molecular weights <1000 Da [99], characterized by high efficacy, low toxicity, strong specificity, and better absorption by the human body [98]. Since KAT8 and its associated H4K16ac are dysregulated in various tumors, small-molecule drugs targeting H4K16ac—including natural compounds, synthetic agents, and non-coding RNA (ncRNA)-based drugs—have demonstrated promising antitumor pharmacological activities and molecular mechanisms.

### 4.1. Natural Compounds Targeting Lysine Acetyltransferase 8 (KAT8)

**Figure 3:**
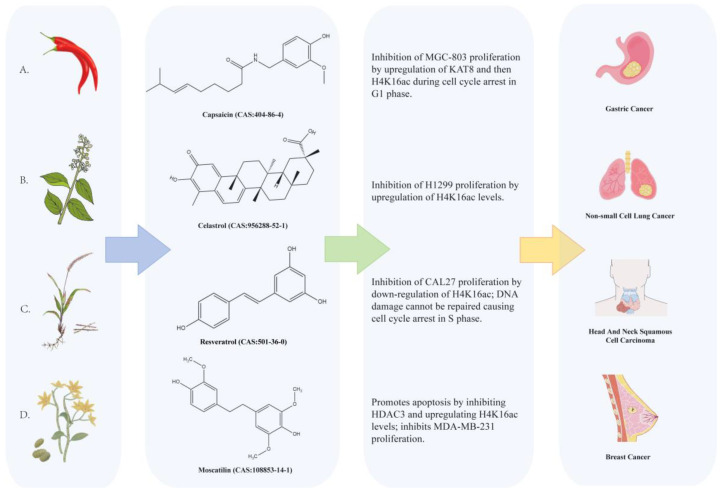
The regulatory mechanism of natural compounds targeting KAT8 in tumors. Source of CAS (https://pubchem.ncbi.nlm.nih.gov) (accessed on 21 May 2025). (**A**): Capsaicin [50]. (**B**): Celastrol [102]. (**C**): Resveratrol [103]. (**D**): Moscatilin [104].

Natural compounds are bioactive molecules extracted from natural sources, with plants being the primary reservoir of anticancer agents [100]. Approximately 30 distinct natural compounds have been isolated from plants to date, and over 3,000 plant-derived compounds are under investigation for cancer therapy and clinical trials [101]. The pharmacological activity and indicated mechanism of natural compounds targeting KAT8 are summarized in Figure 3.

Capsaicin (8-methyl-N-vanillyl-6-noneamide) is an alkaloid extracted from chili pepper resin [100]. Its oral bioavailability ranges from 36% to 40%, and it is primarily metabolized via hepatic and intracerebral pathways [101]. The capsaicin transdermal patch Qutenza (containing 8% capsaicin, 179 mg) is approved by the US Food and Drug Administration (FDA) to treat neuropathic pain from postherpetic neuralgia [105]. Extensive studies have revealed capsaicin’s antitumor effects. For example, it upregulated apoptosis-related proteins (e.g., caspase 3 [*CASP3*] and p53) to induce cell death in NSCLC, bladder cancer, and glioma [106]. Capsaicin also downregulated cyclin D and cyclin E, inducing G1 phase arrest to suppress breast and prostate cancer progression [107]. In MGC-803 gastric cancer cells, capsaicin upregulated KAT8 and enhanced global H4K16ac levels, leading to G1 phase arrest and inhibited proliferation [50]. These findings suggest capsaicin’s potential to suppress tumors with low KAT8 expression.

Celastrol ((2R,4aS,6aS,6aR,14aS,14bR)-10-Hydroxy-2,4a,6a,6a,9,14a-hexamethyl-11-oxo-1,3,4,5,6,13,14,14b-octahydropicene-2-carboxylic acid), a pentacyclic triterpenoid isolated from Tripterygium wilfordii [108], exhibits anti-inflammatory, antitumor, anti-obesity, and neuroprotective activities [109]. Over 60% of its metabolites are excreted via urine, with minor amounts via the intestines, although hepatotoxicity, nephrotoxicity, and reproductive risks remain concerns [110]. Recent studies have highlighted celastrol’s ability to inhibit breast cancer, prostate cancer, and glioma progression [111]. In non-small cell lung cancer (NSCLC) cells, celastrol exerts antitumor effects through dual epigenetic and apoptotic mechanisms. Specifically, celastrol suppresses HDAC1 and HDAC4 expression, consequently elevating global H4K16ac levels. This epigenetic reprogramming activates caspase-3-dependent apoptotic pathways, ultimately inhibiting NSCLC cell proliferation [102]. The pharmacological activity of celastrol in inhibiting the proliferation of NSCLC is also confirmed in vivo. However, its bioavailability (<40%) is limited by gastrointestinal enzymatic degradation and first-pass metabolism, necessitating formulation improvements.

Resveratrol (3,4′,5-trihydroxy-trans-stilbene), a natural antibiotic from Imperata cylindrica, possesses antioxidant, anti-inflammatory, cardioprotective, and anticancer properties [112]. Its plasma concentrations peak within one hour post-administration, and it is metabolized via intestinal and renal pathways [113]. It is demonstrated that resveratrol could inhibit the proliferation of hematologic malignancies, breast, skin, cervical, ovarian, gastric, prostate, colon, liver, pancreatic, and thyroid cancers [114,115,116,117]. For example, resveratrol downregulated Bcl-2 to induce apoptosis in breast cancer cells [118]. It also reduced H4K16ac levels, triggered S-phase arrest via DNA damage activation, and elevated γH2AX levels in head and neck squamous cell carcinoma cells, suppressing proliferation [103].

Moscatilin (4,4′-Dihydroxy-3,3′,5-trimethoxybibenzyl), a bibenzyl derivative from dendrobium species, exhibits immunomodulatory, antioxidant, and anti-ageing effects [119]. Its absorption is limited by gastrointestinal enzymes, gut microbiota metabolism, and first-pass metabolism, with its metabolism occurring in the intestine and liver [120]. Moscatilin inhibited the proliferation of colon, breast, pancreatic, and lung cancer cells [121,122,123]. For example, it upregulated p53 to induce apoptosis in pancreatic cancer cells. It also inhibited HDAC3, elevated H4K16ac levels, and promoted apoptosis in MDA-MB-231 breast cancer cells [104].

### 4.2. Synthetic Small-Molecule Compounds Targeting Lysine Acetyltransferase 8 (KAT8)

Aspirin (2-acetoxybenzoic acid), a salicylate derivative, exhibits high oral bioavailability (~80%), reaching peak plasma concentrations within 0.5–2 h [124]. It is hydrolyzed to salicylic acid by esterases in the gastrointestinal tract, blood, and liver [125]. Salicylate inhibited KAT8 expression, reduced H4K16ac in the mucin 1 (MUC1) promoter, downregulated AKT phosphorylation, and suppressed EMT in gastric cancer cells [126].

Chidamide (N-(2-Amino-4-fluorophenyl)-4-[[(E)-3-pyridin-3-ylprop-2-enoyl]aminomethyl]benzamide), an HDAC inhibitor, had an oral bioavailability of ~70% and a maximum tolerated dose of >5 g/kg in mice. It is approved by China’s FDA for relapsed/refractory peripheral T-cell lymphoma [127]. It upregulated KAT8 and the histone methyltransferase EZH2 in H929 and RPMI8226 myeloma cells, enhancing H4K16ac and H3K27me3 levels in the microtubule-associated protein 1 light chain 3 beta (MAP1LC3B/LC3B) promoter, enhancing LC3B transcription, activating autophagy, and inhibiting myeloma proliferation [128].

**Figure 4:**
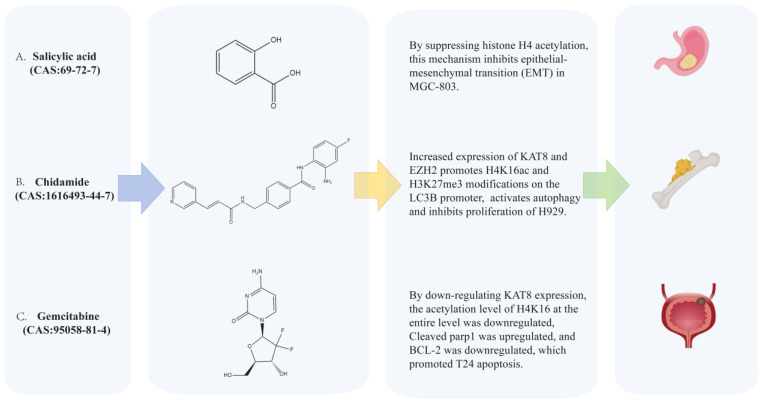
The regulatory mechanism of synthetic small-molecule compounds targeting KAT8 in tumors. Source of CAS (https://pubchem.ncbi.nlm.nih.gov) (accessed on 21 May 2025). (**A**): Salicylic acid [126]. (**B**): Chidamide [128]. (**C**): Gemcitabine [132].

Gemcitabine (4-amino-1-(2-deoxy-2,2-difluoro-β-D-erythro-pentofuranosyl)pyrimidin-2(1H)-one hydrochloride), a pyrimidine nucleoside analogue [129], exhibited ~100% bioavailability (~50% oral bioavailability). Its plasma concentrations peaked at 3.2–45.5 μg/mL 30 min post-infusion, with a half-life of 42–94 min. It is metabolized in the liver and excreted renally [130]. It is FDA-approved for advanced ovarian, breast, NSCLC, and pancreatic cancers [131]. It downregulated KAT8 and H4K16ac levels in T24 bladder cancer cells, upregulated cleaved poly(ADP-ribose) polymerase 1 (PARP1), downregulated BCL2, and promoted apoptosis [132]. The pharmacological activity and indicated mechanism of synthetic small-molecule compounds targeting KAT8 are summarized in Figure 4.

### 4.3. Metal-Based Agents Targeting Lysine Acetyltransferase 8 (KAT8)

Inorganic arsenic, a class I carcinogen, modulates histone post-translational modifications and shows anti-tumor activity in indicated types of cancer. In myeloma cells, arsenic trioxide (As_2_O_3_) enhanced lymphokine-activated killer-mediated cytotoxicity to suppress proliferation [133]. In HeLa cells, As_2_O_3_ directly bounds to KAT8, inactivating its acetyltransferase activity, reducing H4K16ac levels, and upregulating HDAC4 [134]. These findings suggest the As_2_O_3_ potential in targeting tumors overexpressing KAT8 beyond acute myeloid leukemia.

Hexavalent chromium (Cr[VI]) induced Nupr1 overexpression, reduced H4K16ac levels, and triggered cell cycle arrest to inhibit HCC proliferation [135].

### 4.4. Non-Coding RNA

Non-coding RNAs refer to a class of RNA molecules that are not translated into proteins [136]. They exhibit tumor-specific targeting capabilities and demonstrate promising therapeutic potential in cancer treatment. Several ncRNA-based biologics have been approved by the FDA for clinical applications [137]. For example, the microRNA (miR)-29-based therapeutic agent Remlarsen (MRG-201) [138], currently undergoing Phase II clinical trials, showed efficacy in treating skin cancer [139]. Additionally, miR-21 has been clinically applied in gynecological cancers, including ovarian, cervical, and endometrial cancers, as it regulates the downstream target programmed cell death 4 (*PDCD4*) [140].

Notably, miR-203 has been reported to upregulate KAT8 expression, promoting p53 acetylation at K120. This epigenetic modification leads to the downregulation of the anti-apoptotic gene BCL2-like 2 (BCL2L2/BCL-W), ultimately inducing apoptosis in human HCT116 colon cancer cells [141]. Conversely, miR-15a and miR-16-1 downregulated KAT8 expression in chronic lymphocytic leukemia cells, decreasing BCL2 levels and, thereby, tumor cell proliferation [142]. Reduced or absent expression of miR-15 and miR-16-1 was observed in 65 patients with chronic B-lymphocytic leukemia with deleted lymphocytic leukemia 1 (*DLEU1/LEU1*) and 2 (*DLEU2/LEU2*) gene deletions [143]. Based on these findings, we hypothesize that miR-15 and mir-16-1 deficiency or downregulation may attenuate their inhibitory effects on KAT8, thereby upregulating BCL2 expression and promoting leukemogenesis and lymphomagenesis. Furthermore, miR-149-5p-mediated KAT8 suppression reduced global H4K16ac levels in 293/APPsw cells, leading to decreased soluble amyloid beta precursor protein (APP) beta peptide production and potentially attenuating Alzheimer’s disease progression [144]. Notably, KAT8 is significantly upregulated in various malignancies, including thyroid carcinoma, glioblastoma multiforme, oral tongue squamous cell carcinoma, NSCLC, and thymic lymphoma, where it modulates cell proliferation [59]. Therefore, these findings suggest that miR-149-5p-mediated KAT8 downregulation may inhibit tumor growth in these malignancies, although experimental validation is required.

Emerging evidence indicates that long intergenic non-protein coding RNA 2541 (LINC02541/RP11-367G18.1) variant 2 (RP11-367G18.1v2) co-regulates genes enriched in tumor-associated pathways [145]. The YY1 transcription factor (*YY1*) is ubiquitously expressed in mammalian cells and interacts with HATs to activate gene transcription [146]. Mechanistically, the RP11-367G18.1v2-YY1 complex enhanced H4K16 acetylation via EP300 lysine acetyltransferase (EP300), activating the hypoxia-inducible gene solute carrier family 2 member 1 (*SLC2A1/GLUT1*). Colony formation assays confirm that upregulation of the YY1 complex suppresses tumorigenicity [147]. In pancreatic β-cells, RP11-367G18.1 promoted EMT by increasing H4K16ac levels in the twist family bHLH transcription factor 1 (*TWIST1*), snail family transcriptional repressor 2 (*SNAI2/SLUG*), and vascular endothelial growth factor A (*VEGFA/VEGF*) promoters by interacting with EP300 [148]. Additionally, circMYO10, a circular RNA derived from myosin X (MYO10) back-splicing, is upregulated in osteosarcoma cell lines [149]. CircMYO10 has been shown to activate Wnt/CTNNB1 signaling via the miR-370-3p/RuvB-like AAA ATPase 1 (RUVBL1) axis and histone modifier lysine acetyltransferase 5 (KAT5/TIP60), increasing H4K16ac levels in the MYC proto-oncogene bHLH transcription factor (*MYC/c-Myc*) promoter to inhibit osteosarcoma progression [150].

## 5. Conclusions and Perspectives

Tumor development and progression are closely associated with dysregulated acetylation homeostasis. While the role of HDACs in oncogenesis is well-established, HAT inhibitors have emerged as promising anticancer therapeutic strategies. Over 20 HAT inhibitors are currently undergoing preclinical and clinical trials as monotherapies or combination therapies, including Zolinza and Istodax, which are approved by the FDA to treat cutaneous T-cell lymphoma. This review focused on the HAT KAT8, systematically summarizing its expression patterns and associated H4K16ac levels across cancers. It elucidated the KAT8 dual regulatory roles in tumorigenesis and progression based on its upregulation or downregulation and its associated signaling pathways. It also catalogued the KAT8-targeting small molecules (natural compounds and synthetic drugs) that modulate tumor cell proliferation by correcting KAT8 and H4K16ac levels and regulating downstream pathways. Notably, while some compounds remain in the exploratory stages and require optimization of their bioactivity and bioavailability before Phase I/II trials, others (e.g., resveratrol) have entered clinical testing or received FDA approval (e.g., gemcitabine). Natural compounds are gaining attention in chemotherapeutic development due to their low toxicity profiles, although challenges in pharmacokinetics and solubility necessitate further refinement.

## Figures and Tables

**Table 1:** Mechanisms of KAT8 in regulating the process of various tumors.

Mechanism	Target	Model	Reference
Apoptosis	KAT8 promotes SIRT1 expression to downregulate STAT3 expression to promote apoptosis.	HCC (HepG2)	[41]
	KAT8 overexpression H4K16 acetylation level upregulation CA9 expression reduction promotes apoptosis.	RCC (786-0)	[36]
	KAT8 interacts with PKM2 and acetylates the PKM2 K433 site thereby upregulating Bcl-2 to restrain apoptosis.	PRAD (BHP10-3, TT2609)	[47]
Cell Proliferation	KAT8 promotes upregulation of H4K16 acetylation levels, cell cycle arrest in G1 phase, and inhibition of cell proliferation.	STAD (MGC-803)	[52]
	Knockdown of KAT8 inhibits tumorigenicity of UM1 cells by downregulating EZH2 expression.	TSCC (UM1)	[43]
	Knockdown of KAT8 downregulates acetylation of NRF2 588 site and inhibits NQO1 and HO-1 expression to suppress cell proliferation.	NSCLC (H1975)	[58]
	KAT8 mediates SIRT6 acetylation to inhibit SIRT6 and FOXA2 interactions, which in turn activates ZEB2 transcription, thereby promoting cell proliferation.	NSCLC (A549, H1299)	[59]
	KAT8 catalyzes the Skp2 promoter region H4K16ac, upregulates p27, promotes cell passage through the S phase, and inhibits cell proliferation.	NSCLC (A549)	[13]
DNA Damage and Repair	KAT8 interacts with PCNA and ubiquitinates it to promote its recruitment to replication stress DNA damage sites for DNA damage repair.	Osteosarcoma (U2OS)	[71]
Autophagy	Downregulation of H4K16 acetylation levels by KAT8 promotes increased LC3-LC3II conversion and activates cellular autophagy.	NSCLC (U1810)	[79]
	KAT8 downregulates H4K16 acetylation levels, PINK1 expression, inhibits p62 recruitment, and inhibits the mitochondrial-lysosomal autophagy pathway.	Renal cell carcinoma (ACNH)	[87]
Invasion and Migration EMT	KAT8 catalyzes the acetylation of the bundle protein Fascin-K41 site and promotes cell invasiveness.	ESCC (KYSE150)	[91]
	KAT8 acetylates the WSTF-K426 site and promotes WSTF-S158 phosphorylation, cell migration, and invasion ability.	Breast cancer (MDAMB-237)	[95]
	KAT8 inhibits LSD1 recruitment at the promoter region and restores the methylation of H3K4 and KRT8, and inhibits EMT and invasive capability.	NSCLC (A549)	[98]

HCC: human hepatocellular carcinoma; PRAD: prostate adenocarcinoma; STAD: stomach adenocarcinoma; TSCC: tongue squamous cell carcinoma; NSCLC: non-small cell lung carcinoma; ESCC: esophageal squamous cell carcinoma.

## References

[1] Kiri S., Ryba T. (2024). Cancer, Metastasis, and the Epigenome. Mol. Cancer.

[2] Zhou R., Tang X., Wang Y. (2024). Emerging Strategies to Investigate the Biology of Early Cancer. Nat. Rev. Cancer.

[3] Zhang J., Wu Y., Li Y., Li S., Liu J., Yang X., Xia G., Wang G. (2024). Natural Products and Derivatives for Breast Cancer Treatment: From Drug Discovery to Molecular Mechanism. Phytomedicine.

[4] Rea S., Xouri G., Akhtar A. (2007). Males Absent on the First (MOF): From Flies to Humans. Oncogene.

[5] Xuan H., Xu L., Li K., Xuan F., Xu T., Wen H., Shi X. (2024). Hotspot Cancer Mutation Impairs KAT8-Mediated Nucleosomal Histone Acetylation. J. Mol. Biol..

[6] An H.-M., Dai Y.-F., Zhu J., Liu W., Wang X.-P. (2024). MYST Family Histone Acetyltransferases Regulate Reproductive Diapause Initiation. Int. J. Biol. Macromol..

[7] Chen C., Pawley S.B., Cote J.M., Carter J., Wang M., Xu C., Buesking A.W. (2024). Identification of Triazolyl KAT6 Inhibitors via a Templated Fragment Approach. Bioorganic Med. Chem. Lett..

[8] Yang Y., Han X., Guan J., Li X. (2014). Regulation and Function of Histone Acetyltransferase MOF. Front. Med..

[9] Chatterjee A., Seyfferth J., Lucci J., Gilsbach R., Preissl S., Böttinger L., Mårtensson C.U., Panhale A., Stehle T., Kretz O. (2016). MOF Acetyl Transferase Regulates Transcription and Respiration in Mitochondria. Cell.

[10] Zhao L., Wang D.-L., Liu Y., Chen S., Sun F.-L. (2013). Histone Acetyltransferase hMOF Promotes S Phase Entry and Tumorigenesis in Lung Cancer. Cell. Signal..

[11] Yoo L., Mendoza D., Richard A.J., Stephens J.M. (2024). KAT8 beyond Acetylation: A Survey of Its Epigenetic Regulation, Genetic Variability, and Implications for Human Health. Genes.

[12] Karoutas A., Szymanski W., Rausch T., Guhathakurta S., Rog-Zielinska E.A., Peyronnet R., Seyfferth J., Chen H.-R., De Leeuw R., Herquel B. (2019). The NSL Complex Maintains Nuclear Architecture Stability via Lamin A/C Acetylation. Nat. Cell Biol..

[13] Wei S., Liu W., Sun N., Wu Y., Song H., Wang C., Wang S., Zou R., Lin L., Zeng K. (2021). MOF Upregulates the Estrogen Receptor α Signaling Pathway by Its Acetylase Activity in Hepatocellular Carcinoma. Cancer Sci..

[14] Nie Q., Huan X., Kang J., Yin J., Zhao J., Li Y., Zhang Z. (2022). MG149 Inhibits MOF-Mediated P53 Acetylation to Attenuate X-Ray Radiation-Induced Apoptosis in H9c2 Cells. Radiat. Res..

[15] Xie M., Zhou L., Li T., Lin Y., Zhang R., Zheng X., Zeng C., Zheng L., Zhong L., Huang X. (2024). Targeting the KAT8/YEATS4 Axis Represses Tumor Growth and Increases Cisplatin Sensitivity in Bladder Cancer. Adv. Sci..

[16] Xian Q., Song Y., Gui C., Zhou Y. (2023). Mechanistic Insights into Genomic Structure and Functions of a Novel Oncogene YEATS4. Front. Cell Dev. Biol..

[17] Hariharan S., Dharmaraj S. (2020). Selenium and Selenoproteins: It’s Role in Regulation of Inflammation. Inflammopharmacology.

[18] Zhu Z., Nie G., Peng X., Zhan X., Ding D. (2025). KAT8 Catalyzes the Acetylation of SEPP1 at Lysine 247/249 and Modulates the Activity of CD8+ T Cells via LRP8 to Promote Anti-Tumor Immunity in Pancreatic Cancer. Cell Biosci..

[19] Dong Z., Zou J., Li J., Pang Y., Liu Y., Deng C., Chen F., Cui H. (2019). MYST1/KAT8 Contributes to Tumor Progression by Activating EGFR Signaling in Glioblastoma Cells. Cancer Med..

[20] Liu Y., Du J., Liu X., Wang L., Han Y., Huang C., Liang R., Zheng F., Shi G., Li B. (2021). MG149 Inhibits Histone Acetyltransferase KAT8-Mediated IL-33 Acetylation to Alleviate Allergic Airway Inflammation and Airway Hyperresponsiveness. SIGNAL Transduct. Target. Ther..

[21] Pessoa Rodrigues C., Chatterjee A., Wiese M., Stehle T., Szymanski W., Shvedunova M., Akhtar A. (2021). Histone H4 Lysine 16 Acetylation Controls Central Carbon Metabolism and Diet-Induced Obesity in Mice. Nat. Commun..

[22] Fraga M.F., Ballestar E., Villar-Garea A., Boix-Chornet M., Espada J., Schotta G., Bonaldi T., Haydon C., Ropero S., Petrie K. (2005). Loss of Acetylation at Lys16 and Trimethylation at Lys20 of Histone H4 Is a Common Hallmark of Human Cancer. Nat. Genet..

[23] Song J.S., Kim Y.S., Kim D.K., Park S.I., Jang S.J. (2012). Global Histone Modification Pattern Associated with Recurrence and Disease-free Survival in Non-small Cell Lung Cancer Patients. Pathol. Int..

[24] Zhang S., Sui L., Kong X., Huang R., Li Z. (2023). HDAC6 Decreases H4K16 and α-Tubulin Acetylation during Porcine Oocyte Maturation. Cell Cycle.

[25] Zhao L.-J., Loewenstein P.M., Green M. (2016). The Adenoviral E1A N-Terminal Domain Represses MYC Transcription in Human Cancer Cells by Targeting Both P300 and TRRAP and Inhibiting MYC Promoter Acetylation of H3K18 and H4K16. Genes. Cancer.

[26] Su J., Wang F., Cai Y., Jin J. (2016). The Functional Analysis of Histone Acetyltransferase MOF in Tumorigenesis. Int. J. Mol. Sci..

[27] Pfister S., Rea S., Taipale M., Mendrzyk F., Straub B., Ittrich C., Thuerigen O., Sinn H.P., Akhtar A., Lichter P. (2008). The Histone Acetyltransferase hMOF Is Frequently Downregulated in Primary Breast Carcinoma and Medulloblastoma and Constitutes a Biomarker for Clinical Outcome in Medulloblastoma. Int. J. Cancer.

[28] Cao L., Zhu L., Yang J., Su J., Ni J., Du Y., Liu D., Wang Y., Wang F., Jin J. (2014). Correlation of Low Expression of hMOF with Clinicopathological Features of Colorectal Carcinoma, Gastric Cancer and Renal Cell Carcinoma. Int. J. Oncol..

[29] Bui H.-T., Yamaoka E., Miyano T. (2004). Involvement of Histone H3 (Ser10) Phosphorylation in Chromosome Condensation Without Cdc2 Kinase and Mitogen-Activated Protein Kinase Activation in Pig Oocytes1. Biol. Reprod..

[30] Fullgrabe J., Lynch-Day M.A., Heldring N., Li W., Struijk R.B., Ma Q., Hermanson O., Rosenfeld M.G., Klionsky D.J., Joseph B. (2013). The Histone H4 Lysine 16 Acetyltransferase hMOF Regulates the Outcome of Autophagy. Nature.

[31] Gupta A., Guerin-Peyrou T.G., Sharma G.G., Park C., Agarwal M., Ganju R.K., Pandita S., Choi K., Sukumar S., Pandita R.K. (2008). The Mammalian Ortholog of Drosophila MOF That Acetylates Histone H4 Lysine 16 Is Essential for Embryogenesis and Oncogenesis. Mol. Cell. Biol..

[32] Yin X., Hao Z., Liu Q., Ding R., Chen L., Jin M., Wang S. (2025). Oncolytic Virus Infection Modulates Lysine Acetyltransferase in Gliomas: Comprehensive Analysis and Experimental Validation of KAT8 in Glioma. J. Cell. Mol. Medi.

[33] Zhang J., Liu H., Pan H., Yang Y., Huang G., Yang Y., Zhou W.-P., Pan Z.-Y. (2014). The Histone Acetyltransferase hMOF Suppresses Hepatocellular Carcinoma Growth. Biochem. Biophys. Res. Commun..

[34] Chen D., Kluz T., Fang L., Zhang X., Sun H., Jin C., Costa M. (2016). Hexavalent Chromium (Cr(VI)) Down-Regulates Acetylation of Histone H4 at Lysine 16 through Induction of Stressor Protein Nupr1. PLoS ONE.

[35] Wang Y., Zhang R., Wu D., Lu Z., Sun W., Cai Y., Wang C., Jin J. (2013). Epigenetic Change in Kidney Tumor: Downregulation of Histone Acetyltransferase MYST1 in Human Renal Cell Carcinoma. J. Exp. Clin. Cancer Res..

[36] Sykes S.M., Mellert H.S., Holbert M.A., Li K., Marmorstein R., Lane W.S., McMahon S.B. (2006). Acetylation of the P53 DNA-Binding Domain Regulates Apoptosis Induction. Mol. Cell.

[37] Chen J., Liu D., Chen B., Yang Y., Zhu H., Li D., Liu K., Zhu L., Liu H., Li M. (2023). The Histone Acetyltransferase Mof Regulates Runx2 and Osterix for Osteoblast Differentiation. Cell Tissue Res..

[38] Kapoor-Vazirani P., Kagey J.D., Powell D.R., Vertino P.M. (2008). Role of hMOF-Dependent Histone H4 Lysine 16 Acetylation in the Maintenance of TMS1/ASC Gene Activity. Cancer Res..

[39] Lu L., Li L., Lv X., Wu X.-S., Liu D.-P., Liang C.-C. (2011). Modulations of hMOF Autoacetylation by SIRT1 Regulate hMOF Recruitment and Activities on the Chromatin. Cell Res..

[40] Peng L., Ling H., Yuan Z., Fang B., Bloom G., Fukasawa K., Koomen J., Chen J., Lane W.S., Seto E. (2012). SIRT1 Negatively Regulates the Activities, Functions, and Protein Levels of hMOF and TIP60. Mol. Cell. Biol..

[41] Guo R., Liang Y., Zou B., Li D., Wu Z., Xie F., Zhang X., Li X. (2022). The Histone Acetyltransferase MOF Regulates SIRT1 Expression to Suppress Renal Cell Carcinoma Progression. Front. Oncol..

[42] Jaganathan A., Chaurasia P., Xiao G.-Q., Philizaire M., Lv X., Yao S., Burnstein K.L., Liu D.-P., Levine A.C., Mujtaba S. (2014). Coactivator MYST1 Regulates Nuclear Factor-κB and Androgen Receptor Functions during Proliferation of Prostate Cancer Cells. Mol. Endocrinol..

[43] Yin F., Lan R., Zhang X., Zhu L., Chen F., Xu Z., Liu Y., Ye T., Sun H., Lu F. (2014). LSD1 Regulates Pluripotency of Embryonic Stem/Carcinoma Cells through Histone Deacetylase 1-Mediated Deacetylation of Histone H4 at Lysine 16. Mol. Cell. Biol..

[44] Wu Y., Zhou L., Zou Y., Zhang Y., Zhang M., Xu L., Zheng L., He W., Yu K., Li T. (2023). Disrupting the Phase Separation of KAT8-IRF1 Diminishes PD-L1 Expression and Promotes Antitumor Immunity. Nat. Cancer.

[45] Liang J., Cao R., Wang X., Zhang Y., Wang P., Gao H., Li C., Yang F., Zeng R., Wei P. (2017). Mitochondrial PKM2 Regulates Oxidative Stress-Induced Apoptosis by Stabilizing Bcl2. Cell Res..

[46] Wang D., Zhao C., Xu F., Zhang A., Jin M., Zhang K., Liu L., Hua Q., Zhao J., Liu J. (2021). Cisplatin-Resistant NSCLC Cells Induced by Hypoxia Transmit Resistance to Sensitive Cells through Exosomal PKM2. Theranostics.

[47] Li Z., Lu X., Zhang J., Liu T., Xu M., Liu S., Liang J. (2024). KAT8 Enhances the Resistance of Lung Cancer Cells to Cisplatin by Acetylation of PKM2. Anticancer. Drugs.

[48] Meunier A., Cornet F., Campos M. (2021). Bacterial Cell Proliferation: From Molecules to Cells. FEMS Microbiol. Rev..

[49] Wang F., Zhao J., Liu D., Zhao T., Lu Z., Zhu L., Cao L., Yang J., Jin J., Cai Y. (2016). Capsaicin Reactivates hMOF in Gastric Cancer Cells and Induces Cell Growth Inhibition. Cancer Biol. Ther..

[50] Xu A., Yang X., Zhao J., Kong S., Tang Q., Li X., Qu H., Wang G. (2025). KAT8 Facilitates the Proliferation of Cancer Cells through Enhancing E7 Function in HPV-Associated Cervical Cancer. bioRxiv.

[51] Qi Y., Tan M., Zheng M., Jin S., Wang H., Liu J., Wang P., Nie X., Gao L., Lin B. (2020). Estrogen/Estrogen Receptor Promotes the Proliferation of Endometrial Carcinoma Cells by Enhancing hMOF Expression. Jpn. J. Clin. Oncol..

[52] Kim K.H., Roberts C.W.M. (2016). Targeting EZH2 in Cancer. Nat. Med..

[53] Zhang D., Jiang M., Li P., Laster K.V., Zhao D., Zhi Y., Wei H., Nie W., Gao Y., Wu Q. (2025). CHI-KAT8i5 Suppresses ESCC Tumor Growth by Inhibiting KAT8-Mediated c-Myc Stability. Cell Rep..

[54] Rojo De La Vega M., Chapman E., Zhang D.D. (2018). NRF2 and the Hallmarks of Cancer. Cancer Cell.

[55] Chen Z., Ye X., Tang N., Shen S., Li Z., Niu X., Lu S., Xu L. (2014). The Histone Acetylranseferase h MOF Acetylates Nrf 2 and Regulates Anti-drug Responses in Human Non-small Cell Lung Cancer. Br. J. Pharmacol..

[56] De Coninck S., Berx G., Taghon T., Van Vlierberghe P., Goossens S. (2019). ZEB2 in T-Cells and T-ALL. Adv. Biol. Regul..

[57] Zhao K., Zheng M., Su Z., Ghosh S., Zhang C., Zhong W., Ho J.W.K., Jin G., Zhou Z. (2023). MOF-Mediated Acetylation of SIRT6 Disrupts SIRT6-FOXA2 Interaction and Represses SIRT6 Tumor-Suppressive Function by Upregulating ZEB2 in NSCLC. Cell Rep..

[58] Li D., Yang Y., Chen B., Guo X., Gao S., Wang M., Duan M., Li X. (2020). MOF Regulates TNK2 Transcription Expression to Promote Cell Proliferation in Thyroid Cancer. Front. Pharmacol..

[59] Blum A., Wang P., Zenklusen J.C. (2018). SnapShot: TCGA-Analyzed Tumors. Cell.

[60] Gorgoulis V.G., Vassiliou L.-V.F., Karakaidos P., Zacharatos P., Kotsinas A., Liloglou T., Venere M., DiTullio R.A., Kastrinakis N.G., Levy B. (2005). Activation of the DNA Damage Checkpoint and Genomic Instability in Human Precancerous Lesions. Nature.

[61] Gaillard H., García-Muse T., Aguilera A. (2015). Replication Stress and Cancer. Nat. Rev. Cancer.

[62] Singh M., Bacolla A., Chaudhary S., Hunt C.R., Pandita S., Chauhan R., Gupta A., Tainer J.A., Pandita T.K. (2020). Histone Acetyltransferase MOF Orchestrates Outcomes at the Crossroad of Oncogenesis, DNA Damage Response, Proliferation, and Stem Cell Development. Mol. Cell. Biol..

[63] Valerio D.G., Xu H., Chen C.-W., Hoshii T., Eisold M.E., Delaney C., Cusan M., Deshpande A.J., Huang C.-H., Lujambio A. (2017). Histone Acetyltransferase Activity of MOF Is Required for MLL-AF9 Leukemogenesis. Cancer Res..

[64] Huo D., Chen H., Cheng Y., Song X., Zhang K., Li M.J., Xuan C. (2020). JMJD6 Modulates DNA Damage Response through Downregulating H4K16ac Independently of Its Enzymatic Activity. Cell Death Differ..

[65] Li X., Corsa C.A.S., Pan P.W., Wu L., Ferguson D., Yu X., Min J., Dou Y. (2010). MOF and H4 K16 Acetylation Play Important Roles in DNA Damage Repair by Modulating Recruitment of DNA Damage Repair Protein Mdc1. Mol. Cell. Biol..

[66] Gupta A., Hunt C.R., Hegde M.L., Chakraborty S., Udayakumar D., Horikoshi N., Singh M., Ramnarain D.B., Hittelman W.N., Namjoshi S. (2014). MOF Phosphorylation by ATM Regulates 53BP1-Mediated Double-Strand Break Repair Pathway Choice. Cell Rep..

[67] Gupta A., Sharma G.G., Young C.S.H., Agarwal M., Smith E.R., Paull T.T., Lucchesi J.C., Khanna K.K., Ludwig T., Pandita T.K. (2005). Involvement of Human MOF in ATM Function. Mol. Cell. Biol..

[68] Hoege C., Pfander B., Moldovan G.-L., Pyrowolakis G., Jentsch S. (2002). RAD6-Dependent DNA Repair Is Linked to Modification of PCNA by Ubiquitin and SUMO. Nature.

[69] Singh D.K., Pandita R.K., Singh M., Chakraborty S., Hambarde S., Ramnarain D., Charaka V., Ahmed K.M., Hunt C.R., Pandita T.K. (2018). MOF Suppresses Replication Stress and Contributes to Resolution of Stalled Replication Forks. Mol. Cell. Biol..

[70] Punchihewa C., Inoue A., Hishiki A., Fujikawa Y., Connelly M., Evison B., Shao Y., Heath R., Kuraoka I., Rodrigues P. (2012). Identification of Small Molecule Proliferating Cell Nuclear Antigen (PCNA) Inhibitor That Disrupts Interactions with PIP-Box Proteins and Inhibits DNA Replication. J. Biol. Chem..

[71] Zhang S.-Y., Song X.-Y., Li Y., Ye L.-L., Zhou Q., Yang W.-B. (2020). Tumor-Associated Macrophages: A Promising Target for a Cancer Immunotherapeutic Strategy. Pharmacol. Res..

[72] Li X., He S., Ma B. (2020). Autophagy and Autophagy-Related Proteins in Cancer. Mol. Cancer.

[73] Turco E., Savova A., Gere F., Ferrari L., Romanov J., Schuschnig M., Martens S. (2021). Reconstitution Defines the Roles of P62, NBR1 and TAX1BP1 in Ubiquitin Condensate Formation and Autophagy Initiation. Nat. Commun..

[74] Kocak M., Ezazi Erdi S., Jorba G., Maestro I., Farrés J., Kirkin V., Martinez A., Pless O. (2022). Targeting Autophagy in Disease: Established and New Strategies. Autophagy.

[75] Meyer N., Henkel L., Linder B., Zielke S., Tascher G., Trautmann S., Geisslinger G., Münch C., Fulda S., Tegeder I. (2021). Autophagy Activation, Lipotoxicity and Lysosomal Membrane Permeabilization Synergize to Promote Pimozide- and Loperamide-Induced Glioma Cell Death. Autophagy.

[76] Xu Y., Wan W. (2023). Acetylation in the Regulation of Autophagy. Autophagy.

[77] Zehender A., Li Y.-N., Lin N.-Y., Stefanica A., Nüchel J., Chen C.-W., Hsu H.-H., Zhu H., Ding X., Huang J. (2021). TGFβ Promotes Fibrosis by MYST1-Dependent Epigenetic Regulation of Autophagy. Nat. Commun..

[78] Romao S., Münz C. (2014). LC3-Associated Phagocytosis. Autophagy.

[79] Levine B., Kroemer G. (2019). Biological Functions of Autophagy Genes: A Disease Perspective. Cell.

[80] Berthier A., Seguin S., Sasco A.J., Bobin J.Y., De Laroche G., Datchary J., Saez S., Rodriguez-Lafrasse C., Tolle F., Fraichard A. (2010). High Expression of Gabarapl1 Is Associated with a Better Outcome for Patients with Lymph Node-Positive Breast Cancer. Br. J. Cancer.

[81] Poillet-Perez L., Jacquet M., Hervouet E., Gauthier T., Fraichard A., Borg C., Pallandre J.-R., Gonzalez B.J., Ramdani Y., Boyer-Guittaut M. (2017). GABARAPL1 Tumor Suppressive Function Is Independent of Its Conjugation to Autophagosomes in MCF-7 Breast Cancer Cells. Oncotarget.

[82] Boyer-Guittaut M., Poillet L., Liang Q., Bôle-Richard E., Ouyang X., Benavides G.A., Chakrama F.-Z., Fraichard A., Darley-Usmar V.M., Despouy G. (2014). The Role of GABARAPL1/GEC1 in Autophagic Flux and Mitochondrial Quality Control in MDA-MB-436 Breast Cancer Cells. Autophagy.

[83] Su W., Li S., Chen X., Yin L., Ma P., Ma Y., Su B. (2017). GABARAPL1 Suppresses Metastasis by Counteracting PI3K/Akt Pathway in Prostate Cancer. Oncotarget.

[84] Liu T.-W., Zhao Y.-M., Jin K.-Y., Wang J.-X., Zhao X.-F. (2024). KAT8 Is Upregulated and Recruited to the Promoter of Atg8 by FOXO to Induce H4 Acetylation for Autophagy under 20-Hydroxyecdysone Regulation. J. Biol. Chem..

[85] De Talhouët C., Esteras N., Soutar M.P.M., O’Callaghan B., Plun-Favreau H. (2024). KAT8 Compound Inhibition Inhibits the Initial Steps of PINK1-Dependant Mitophagy. Sci. Rep..

[86] Yan M., Wang J., Wang H., Zhou J., Qi H., Naji Y., Zhao L., Tang Y., Dai Y. (2023). Knockdown of NR3C1 Inhibits the Proliferation and Migration of Clear Cell Renal Cell Carcinoma through Activating Endoplasmic Reticulum Stress-Mitophagy. J. Transl. Med..

[87] Van De Merbel A.F., Van Der Horst G., Buijs J.T., Van Der Pluijm G., Culig Z. (2018). Protocols for Migration and Invasion Studies in Prostate Cancer. Prostate Cancer.

[88] Duff D., Long A. (2017). Roles for RACK1 in Cancer Cell Migration and Invasion. Cell. Signal..

[89] Li D., Cheng Y., Pan J., Guo Z., Wang S., Huang Q., Nie P., Shi W., Xu X., Wen B. (2024). KAT8/SIRT7-mediated Fascin-K41 Acetylation/Deacetylation Regulates Tumor Metastasis in Esophageal Squamous Cell Carcinoma. J. Pathol..

[90] Li P., Yang L., Park S.Y., Liu F., Li A.H., Zhu Y., Sui H., Gao F., Li L., Ye L. (2024). Stabilization of MOF (KAT8) by USP10 Promotes Esophageal Squamous Cell Carcinoma Proliferation and Metastasis through Epigenetic Activation of ANXA2/Wnt Signaling. Oncogene.

[91] Qiu B., Li S., Li M., Wang S., Mu G., Chen K., Wang M., Zhu W., Wang W., Wang J. (2023). KAT8 Acetylation-Controlled Lipolysis Affects the Invasive and Migratory Potential of Colorectal Cancer Cells. Cell Death Dis..

[92] Liu Y., Zhang Y.-Y., Wang S.-Q., Li M., Long Y.-H., Li Y.-F., Liu Y.-K., Li Y.-H., Wang Y.-Q., Mi J.-S. (2020). WSTF Acetylation by MOF Promotes WSTF Activities and Oncogenic Functions. Oncogene.

[93] Nieto M.A., Huang R.Y.-J., Jackson R.A., Thiery J.P. (2016). EMT: 2016. Cell.

[94] Pastushenko I., Blanpain C. (2019). EMT Transition States during Tumor Progression and Metastasis. Trends Cell Biol..

[95] Chen H., Chen X., Pan B., Zheng C., Hong L., Han W. (2022). KRT8 Serves as a Novel Biomarker for LUAD and Promotes Metastasis and EMT via NF-κB Signaling. Front. Oncol..

[96] Luo H., Shenoy A.K., Li X., Jin Y., Jin L., Cai Q., Tang M., Liu Y., Chen H., Reisman D. (2016). MOF Acetylates the Histone Demethylase LSD1 to Suppress Epithelial-to-Mesenchymal Transition. Cell Rep..

[97] Zhang J., Yang P.L., Gray N.S. (2009). Targeting Cancer with Small Molecule Kinase Inhibitors. Nat. Rev. Cancer.

[98] Roy A. (2021). Plumbagin: A Potential Anti-Cancer Compound. Mini Rev. Med. Chem..

[99] Choo M.Z.Y., Chua J.A.T., Lee S.X.Y., Ang Y., Wong W.S.F., Chai C.L.L. (2025). Privileged Natural Product Compound Classes for Anti-Inflammatory Drug Development. Nat. Prod. Rep..

[100] Kim J.-D., Kim J.-M., Pyo J.-O., Kim S.-Y., Kim B.-S., Yu R., Han I.-S. (1997). Capsaicin Can Alter the Expression of Tumor Forming-Related Genes Which Might Be Followed by Induction of Apoptosis of a Korean Stomach Cancer Cell Line, SNU-1. Cancer Lett..

[101] Frias B., Merighi A. (2016). Capsaicin, Nociception and Pain. Molecules.

[102] Hou W., Liu B., Xu H. (2020). Celastrol: Progresses in Structure-Modifications, Structure-Activity Relationships, Pharmacology and Toxicology. Eur. J. Med. Chem..

[103] Contreras S.M., Ganuza A., Corvi M.M., Angel S.O. (2021). Resveratrol Induces H3 and H4K16 Deacetylation and H2A.X Phosphorylation in *Toxoplasma gondii*. BMC Res. Notes.

[104] Lai M.C., Liu W.Y., Liou S.-S., Liu I.-M. (2020). The Protective Effects of Moscatilin against Methylglyoxal-Induced Neurotoxicity via the Regulation of P38/JNK MAPK Pathways in PC12 Neuron-like Cells. Food Chem. Toxicol..

[105] Wallace M., Pappagallo M. (2011). Qutenza^®^: A Capsaicin 8% Patch for the Management of Postherpetic Neuralgia. Expert. Rev. Neurother..

[106] Wang H.-M., Chuang S.-M., Su Y.-C., Li Y.-H., Chueh P.J. (2011). Down-Regulation of Tumor-Associated NADH Oxidase, tNOX (ENOX2), Enhances Capsaicin-Induced Inhibition of Gastric Cancer Cell Growth. Cell Biochem. Biophys..

[107] Thoennissen N.H., O’Kelly J., Lu D., Iwanski G.B., La D.T., Abbassi S., Leiter A., Karlan B., Mehta R., Koeffler H.P. (2010). Capsaicin Causes Cell-Cycle Arrest and Apoptosis in ER-Positive and -Negative Breast Cancer Cells by Modulating the EGFR/HER-2 Pathway. Oncogene.

[108] Wang C., Dai S., Zhao X., Zhang Y., Gong L., Fu K., Ma C., Peng C., Li Y. (2023). Celastrol as an Emerging Anticancer Agent: Current Status, Challenges and Therapeutic Strategies. Biomed. Pharmacother..

[109] Sun Y., Wang C., Li X., Lu J., Wang M. (2024). Recent Advances in Drug Delivery of Celastrol for Enhancing Efficiency and Reducing the Toxicity. Front. Pharmacol..

[110] Kashyap D., Sharma A., Tuli H.S., Sak K., Mukherjee T., Bishayee A. (2018). Molecular Targets of Celastrol in Cancer: Recent Trends and Advancements. Crit. Rev. Oncol. Hematol..

[111] Chen G., Zhu X., Li J., Zhang Y., Wang X., Zhang R., Qin X., Chen X., Wang J., Liao W. (2022). Celastrol Inhibits Lung Cancer Growth by Triggering Histone Acetylation and Acting Synergically with HDAC Inhibitors. Pharmacol. Res..

[112] Ren B., Kwah M.X.-Y., Liu C., Ma Z., Shanmugam M.K., Ding L., Xiang X., Ho P.C.-L., Wang L., Ong P.S. (2021). Resveratrol for Cancer Therapy: Challenges and Future Perspectives. Cancer Lett..

[113] Luca S.V., Macovei I., Bujor A., Miron A., Skalicka-Woźniak K., Aprotosoaie A.C., Trifan A. (2020). Bioactivity of Dietary Polyphenols: The Role of Metabolites. Crit. Rev. Food Sci. Nutr..

[114] Aggarwal B.B., Bhardwaj A., Aggarwal R.S., Seeram N.P., Shishodia S., Takada Y. (2004). Role of Resveratrol in Prevention and Therapy of Cancer: Preclinical and Clinical Studies. Anticancer. Res..

[115] Harikumar K.B., Kunnumakkara A.B., Sethi G., Diagaradjane P., Anand P., Pandey M.K., Gelovani J., Krishnan S., Guha S., Aggarwal B.B. (2010). Resveratrol, a Multitargeted Agent, Can Enhance Antitumor Activity of Gemcitabine in Vitro and in Orthotopic Mouse Model of Human Pancreatic Cancer. Intl J. Cancer.

[116] Tomé-Carneiro J., Larrosa M., González-Sarrías A., Tomás-Barberán F., García-Conesa M., Espín J. (2013). Resveratrol and Clinical Trials: The Crossroad from In Vitro Studies to Human Evidence. Curr. Pharm. Des..

[117] Kundu J.K., Surh Y.-J. (2008). Cancer Chemopreventive and Therapeutic Potential of Resveratrol: Mechanistic Perspectives. Cancer Lett..

[118] Auti A., Alessio N., Ballini A., Dioguardi M., Cantore S., Scacco S., Vitiello A., Quagliuolo L., Rinaldi B., Santacroce L. (2022). Protective Effect of Resveratrol against Hypoxia-Induced Neural Oxidative Stress. J. Pers. Med..

[119] Xiong L., Cao Z.-X., Peng C., Li X.-H., Xie X.-F., Zhang T.-M., Zhou Q.-M., Yang L., Guo L. (2013). Phenolic Glucosides from *Dendrobium aurantiacum* Var. Denneanum and Their Bioactivities. Molecules.

[120] Silva-Reis R., Silva V.L.M., Cardoso S.M., Michalak I., Püsküllüoğlu M., Calina D., Sharifi-Rad J. (2024). Moscatilin, a Potential Therapeutic Agent for Cancer Treatment: Insights into Molecular Mechanisms and Clinical Prospects. Med. Oncol..

[121] Chen T.-H., Pan S.-L., Guh J.-H., Liao C.-H., Huang D.-Y., Chen C.-C., Teng C.-M. (2008). Moscatilin Induces Apoptosis in Human Colorectal Cancer Cells: A Crucial Role of c-Jun NH2-Terminal Protein Kinase Activation Caused by Tubulin Depolymerization and DNA Damage. Clin. Cancer Res..

[122] Zhang L., Fang Y., Xu X.-F., Jin D.-Y. (2017). Moscatilin Induces Apoptosis of Pancreatic Cancer Cells via Reactive Oxygen Species and the JNK/SAPK Pathway. Mol. Med. Rep..

[123] Su W., Zeng L., Chen W. (2021). Moscatilin Suppresses the Breast Cancer Both In Vitro and In Vivo by Inhibiting HDAC3. Dose-Response.

[124] Nelson M.R., Black J.A. (2024). Aspirin: Latest Evidence and Developments. Heart.

[125] Lichtenberger L.M., Phan T., Fang D., Edler S., Philip J., Li-Geng T., Dial E.J. (2016). Bioavailability of Aspirin in Rats Comparing the Drug’s Uptake into Gastrointestinal Tissue and Vascular and Lymphatic Systems: Implications on Aspirin’s Chemopreventive Action. J. Physiol. Pharmacol..

[126] Fernandez H.R., Lindén S.K. (2017). The Aspirin Metabolite Salicylate Inhibits Lysine Acetyltransferases and MUC1 Induced Epithelial to Mesenchymal Transition. Sci. Rep..

[127] Chan T., Tse E., Kwong Y.-L. (2017). Chidamide in the Treatment of Peripheral T-Cell Lymphoma. OncoTargets Ther..

[128] Xu L., Feng J., Tang H., Dong Y., Shu M., Chen X. (2020). Chidamide Epigenetically Represses Autophagy and Exerts Cooperative Antimyeloma Activity with Bortezomib. Cell Death Dis..

[129] Miao H., Chen X., Luan Y. (2020). Small Molecular Gemcitabine Prodrugs for Cancer Therapy. Curr. Med. Chem..

[130] Thompson B.R., Shi J., Zhu H.-J., Smith D.E. (2020). Pharmacokinetics of Gemcitabine and Its Amino Acid Ester Prodrug Following Intravenous and Oral Administrations in Mice. Biochem. Pharmacol..

[131] Wang J., Yang J., Narang A., He J., Wolfgang C., Li K., Zheng L. (2024). Consensus, Debate, and Prospective on Pancreatic Cancer Treatments. J. Hematol. Oncol..

[132] Zhu H., Wang Y., Wei T., Zhao X., Li F., Li Y., Wang F., Cai Y., Jin J. (2021). KAT8/MOF-Mediated Anti-Cancer Mechanism of Gemcitabine in Human Bladder Cancer Cells. Biomol. Ther..

[133] Howe C.G., Gamble M.V. (2016). Influence of Arsenic on Global Levels of Histone Posttranslational Modifications: A Review of the Literature and Challenges in the Field. Curr. Environ. Health Rep..

[134] Chen J., Jin Z., Zhang S., Zhang X., Li P., Yang H., Ma Y. (2022). Arsenic Trioxide Elicits Prophylactic and Therapeutic Immune Responses against Solid Tumors by Inducing Necroptosis and Ferroptosis. Cell Mol. Immunol..

[135] Ma P., Schultz R.M. (2013). Histone Deacetylase 2 (HDAC2) Regulates Chromosome Segregation and Kinetochore Function via H4K16 Deacetylation during Oocyte Maturation in Mouse. PLoS Genet..

[136] Guo X., Wang W., Hu J., Feng K., Pan Y., Zhang L., Feng Y. (2012). Lentivirus-Mediated RNAi Knockdown of NUPR1 Inhibits Human Nonsmall Cell Lung Cancer Growth In Vitro and In Vivo. Anat. Rec..

[137] Chan J.J., Tay Y. (2018). Noncoding RNA:RNA Regulatory Networks in Cancer. Int. J. Mol. Sci..

[138] Winkle M., El-Daly S.M., Fabbri M., Calin G.A. (2021). Noncoding RNA Therapeutics—Challenges and Potential Solutions. Nat. Rev. Drug Discov..

[139] Gallant-Behm C.L., Piper J., Lynch J.M., Seto A.G., Hong S.J., Mustoe T.A., Maari C., Pestano L.A., Dalby C.M., Jackson A.L. (2019). A MicroRNA-29 Mimic (Remlarsen) Represses Extracellular Matrix Expression and Fibroplasia in the Skin. J. Investig. Dermatol..

[140] Murata A., Otabe T., Zhang J., Nakatani K. (2016). BzDANP, a Small-Molecule Modulator of Pre-miR-29a Maturation by Dicer. ACS Chem. Biol..

[141] Jiang N.-J., Yin Y.-N., Lin J., Li W.-Y., Long D.-R., Mei L. (2023). MicroRNA-21 in Gynecological Cancers: From Molecular Pathogenesis to Clinical Significance. Pathol.-Res. Pract..

[142] Chang J., Davis-Dusenbery B.N., Kashima R., Jiang X., Marathe N., Sessa R., Louie J., Gu W., Lagna G., Hata A. (2013). Acetylation of P53 Stimulates miRNA Processing and Determines Cell Survival Following Genotoxic Stress: Acetylation of P53 Stimulates miRNA Processing and Determines Cell Survival. EMBO J..

[143] Calin G.A., Ferracin M., Cimmino A., Di Leva G., Shimizu M., Wojcik S.E., Iorio M.V., Visone R., Sever N.I., Fabbri M. (2005). A MicroRNA Signature Associated with Prognosis and Progression in Chronic Lymphocytic Leukemia. N. Engl. J. Med..

[144] Calin G.A., Dumitru C.D., Shimizu M., Bichi R., Zupo S., Noch E., Aldler H., Rattan S., Keating M., Rai K. (2002). Frequent Deletions and Down-Regulation of Micro- RNA Genes miR15 and miR16 at 13q14 in Chronic Lymphocytic Leukemia. Proc. Natl. Acad. Sci. USA.

[145] Chen F., Chen H., Jia Y., Lu H., Tan Q., Zhou X. (2020). miR-149-5p Inhibition Reduces Alzheimer’s Disease &beta;-amyloid Generation in 293/APPsw Cells by Upregulating H4K16ac via KAT8. Exp. Ther. Med..

[146] Shao I., Peng P., Wu H., Chen J., Lai J.C., Chang J., Wu H., Wu K., Pang S., Hsu K. (2023). RP11-367G18.1 V2 Enhances Clear Cell Renal Cell Carcinoma Progression via Induction of Epithelial–Mesenchymal Transition. Cancer Med..

[147] Verheul T.C.J., Van Hijfte L., Perenthaler E., Barakat T.S. (2020). The Why of YY1: Mechanisms of Transcriptional Regulation by Yin Yang 1. Front. Cell Dev. Biol..

[148] Peng P.-H., Chen J.-L., Wu H.-H., Yang W.-H., Lin L.-J., Lai J.C.-Y., Chang J.-S., Syu J.-L., Wu H.-T., Hsu F.-T. (2023). Interplay between lncRNA RP11-367G18.1 Variant 2 and YY1 Plays a Vital Role in Hypoxia-Mediated Gene Expression and Tumorigenesis. Cancer Cell Int..

[149] Liu G., Huang K., Jie Z., Wu Y., Chen J., Chen Z., Fang X., Shen S. (2018). CircFAT1 Sponges miR-375 to Promote the Expression of Yes-Associated Protein 1 in Osteosarcoma Cells. Mol. Cancer.

[150] Chen J., Liu G., Wu Y., Ma J., Wu H., Xie Z., Chen S., Yang Y., Wang S., Shen P. (2019). CircMYO10 Promotes Osteosarcoma Progression by Regulating miR-370-3p/RUVBL1 Axis to Enhance the Transcriptional Activity of β-Catenin/LEF1 Complex via Effects on Chromatin Remodeling. Mol. Cancer.

